# Climacostol reduces tumour progression in a mouse model of melanoma via the p53-dependent intrinsic apoptotic programme

**DOI:** 10.1038/srep27281

**Published:** 2016-06-07

**Authors:** Cristiana Perrotta, Federico Buonanno, Silvia Zecchini, Alessio Giavazzi, Francesca Proietti Serafini, Elisabetta Catalani, Laura Guerra, Maria Cristina Belardinelli, Simona Picchietti, Anna Maria Fausto, Simone Giorgi, Enrico Marcantoni, Emilio Clementi, Claudio Ortenzi, Davide Cervia

**Affiliations:** 1 Department of Biomedical and Clinical Sciences “Luigi Sacco” (DIBIC), Università degli Studi di Milano, Italy; 2 Laboratory of Protistology and Biology Education, Department of Education, Cultural Heritage and Tourism, Università degli Studi di Macerata, Italy; 3 Unit of Clinical Pharmacology, University Hospital “Luigi Sacco”-ASST Fatebenefratelli Sacco, Milano, Italy; 4 Department for Innovation in Biological, Agro-food and Forest systems (DIBAF), Università degli Studi della Tuscia, Viterbo, Italy; 5 School of Sciences and Technologies, Section of Chemistry, Università degli Studi di Camerino, Italy; 6 Unit of Clinical Pharmacology, University Hospital “Luigi Sacco”-ASST Fatebenefratelli Sacco; National Research Council-Institute of Neuroscience; Department of Biomedical and Clinical Sciences “Luigi Sacco” (DIBIC), Università degli Studi di Milano, Italy; 7 Scientific Institute IRCCS Eugenio Medea, Bosisio Parini, Italy

## Abstract

Climacostol, a compound produced by the ciliated protozoan *Climacostomum virens*, displayed cytotoxic properties *in vitro*. This study demonstrates that it has anti-tumour potential. Climacostol caused a reduction of viability/proliferation of B16-F10 mouse melanoma cells, a rapidly occurring DNA damage, and induced the intrinsic apoptotic pathway characterised by the dissipation of the mitochondrial membrane potential, the translocation of Bax to the mitochondria, the release of Cytochrome c from the mitochondria, and the activation of Caspase 9-dependent cleavage of Caspase 3. The apoptotic mechanism of climacostol was found to rely on the up-regulation of p53 and its targets Noxa and Puma. *In vivo* analysis of B16-F10 allografts revealed a persistent inhibition of tumour growth rate when melanomas were treated with intra-tumoural injections of climacostol. In addition, it significantly improved the survival of transplanted mice, decreased tumour weight, induced a remarkable reduction of viable cells inside the tumour, activated apoptosis and up-regulated the p53 signalling network. Importantly, climacostol toxicity was more selective against tumour than non-tumour cells. The anti-tumour properties of climacostol and the molecular events associated with its action indicate that it is a powerful agent that may be considered for the design of pro-apoptotic drugs for melanoma therapy.

The incidence of melanoma skin cancer has risen sharply over past few decades, making melanoma one of the commonest tumours in Caucasian worldwide^1^,^2^. Presently the standard chemotherapy in the treatment of unresectable/metastatic melanoma is largely palliative^3^. Multiple new targeted therapies and immunotherapies have been recently approved for advanced melanoma which have supplanted chemotherapy as first- and second-line therapy^4^,^5^,^6^. Despite the new advances seen in melanoma treatment, cure for most melanomas is still elusive and patients have to undergo several lines of therapy alone or in combination to maximise their chances of survival^4^,^5^,^6^,^7^. Since melanoma cells eventually become resistant to anti-cancer compounds and are able to circumvent their effects, the quest for novel molecules which may improve (alone or in combination therapy) the treatment currently in use is still open and of relevance in clinical perspective^3^,^4^. An abundance of natural resources for medicinal use are known worldwide, of which many have not yet been exploited for possible application in the pharmaceutical industry. Over 70% of anti-cancer agents have their origin in natural sources and, in particular, natural products provide a variety of lead compounds used or currently under investigations for their anti-melanoma activities^3^,^8^,^9^.

Climacostol (1,3-dihydroxy-5-[(Z)-non-2′-enyl]benzene) is a natural toxin physiologically produced by the freshwater ciliated protozoan *Climacostomum virens* for chemical defence against unicellular^10^ and multi-cellular predators^11^. The toxin belongs to resorcinolic lipids, a large group of natural amphiphilic compounds, widely detected in prokaryotes and eukaryotes, that have attracted attention for their anti-microbial, anti-parasitic, anti-tumour and genotoxic activities^12^.

Climacostol, now available by a new and straightforward synthesis^13^, displayed potent cytotoxic activity on a panel of bacterial and fungal pathogens, as well as on free-living freshwater ciliates^14^,^15^. Preliminary findings also demonstrated that climacostol inhibits the growth of tumour cells and induces apoptosis *in vitro*^12^,^13^. However, the signalling events and the anti-neoplastic activity of climacostol have never been elucidated.

In this study, using multiple tumour and non-tumour cell line models the anti-tumour properties of climacostol has been characterised. In particular, we investigated the cytotoxic, anti-proliferative and pro-apoptotic effects of climacostol and the molecular signature associated with its action on melanoma cells. The first proof of concept that climacostol has therapeutic activity *in vivo* was also provided by means of a melanoma allograft model. These results give a more exhaustive picture of the toxin activity spectrum and represent a valuable clue for the development of climacostol-based strategy for melanoma therapy.

## Results and Discussion

### Climacostol inhibits tumour cell growth

**Figure 1:**
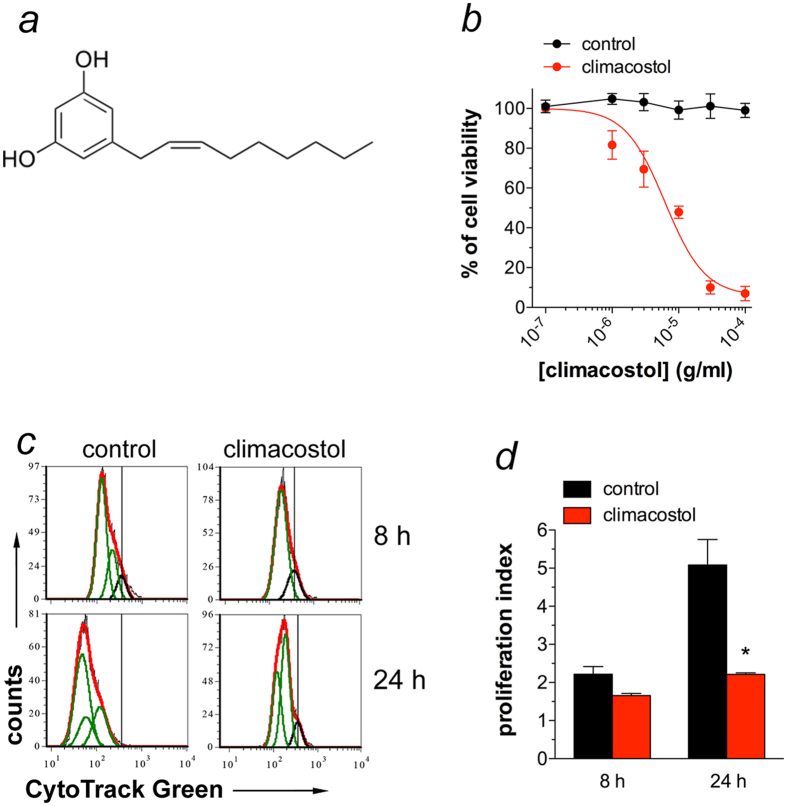
Cytotoxic and anti-proliferative properties of climacostol in melanoma cells. (**a**) The chemical structure of climacostol. (**b**) B16-F10 cells were treated with increasing concentrations of climacostol for 24 h before the MTT assay. Serial dilutions of vehicle (control) were also used. Data are expressed by setting the absorbance of the reduced MTT in the absence of climacostol as 100%. The data points represent the results obtained from 4 independent experiments. (**c**) Flow cytometry analysis of B16-F10 proliferation at 8 and 24 h after cell treatment with climacostol (30 μg/ml) or vehicle (control). The vertical black line represents the undivided cell peak used as point of reference for the CytoTrack Green profile. The data are representative of 3 independent experiments. (**d**) Proliferation index for experiments shown in (**c**). *p < 0.001 *vs* the respective control.

Climacostol (Fig. 1a) has been shown to decrease the growth of different human tumour cells while being devoid of effects on endothelial cells^12^,^13^. These data have been now expanded using a panel of additional cells of tumour and non-tumour origin confirming the pharmacological properties and efficacy of this drug. Cells were analysed by Lactate dehydrogenase (LDH) activity and 3-(4,5-dimethylthiazol-2-yl)-2,5-diphenyltetrazolium bromide (MTT) assay after the treatment with climacostol at increasing concentrations for 24 h. As shown in Table 1, climacostol decreased MTT absorbance of Hela, P3X, PC12, and AtT-20 cells with an EC_50_ ranging from 0.60 to 6.23 μg/ml; instead, the EC_50_ values obtained analysing TM3 and NIH/3T3 cell viability were more than 11.30 μg/ml. These comparative data indicated that viability of tumour cells is negatively affected by climacostol with higher potency when compared to non-tumour cells.

### Climacostol inhibits viability and proliferation of melanoma cells

The cytotoxic properties of climacostol were then examined on the highly tumourigenic B16 mouse melanoma cells, which is a well-used model in melanoma research^16^,^17^,^18^. In line with results obtained in other tumour cells, B16-F10 cell treatment with climacostol (24 h) caused a concentration-dependent reduction of MTT absorbance with an EC_50_ of 6.23 μg/ml and an E_max_ concentration value of 30 μg/ml (Fig. 1b). The E_max_ concentration of climacostol decreased cell viability by nearly 90%.

Deregulated cell proliferation is a hallmark of cancer. To assess whether the climacostol-induced inhibition of cell viability impacts on melanoma proliferation rate, B16-F10 cells were analysed by flow cytometry at a single-cell level. As shown in Fig. 1c, cell proliferation was clearly inhibited during 24 h of climacostol (30 μg/ml) exposure while no difference was observed at 8 h. In particular, the cell proliferation index in the presence of 24 h climacostol significantly decreased by 57% when compared to control (2.21 ± 0.04 *vs* 5.08 ± 0.67 in vehicle-treated cells) (Fig. 1d). These results demonstrate the potent and effective cytotoxic and anti-proliferative role of climacostol in melanoma cells.

### Climacostol induces a fast DNA damage in melanoma cells and does not display additive effects with the chemotherapeutic compound cisplatin

**Figure 2:**
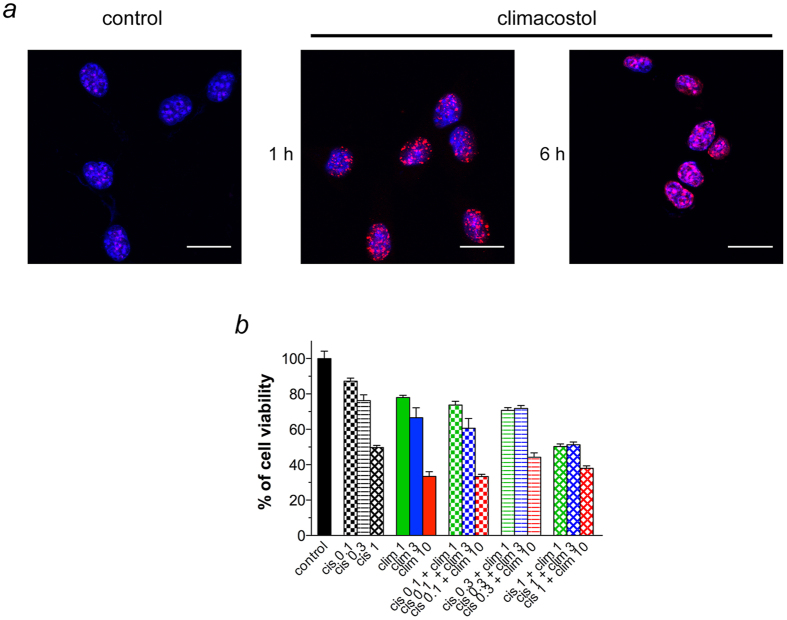
DNA damaging effects of climacostol in melanoma cells and absence of additive effects with DNA binding agents. (**a**) Confocal immunofluorescence imaging of phospho-histone H2A.X in B16-F10 cells cultured in the presence of 30 μg/ml climacostol or vehicle (control) for 1 or 6 h. DAPI (blue) was used for nuclei detection. The images are representative of 4 independent experiments. Scale bar: 20 μm. (**b**) MTT assay of B16-F10 cells treated for 24 h in the absence (control) or in the presence of increasing concentrations of cisplatin (0.1, 0.3, and 1 μg/ml) and climacostol (1, 3, and 10 μg/ml). Compounds were administered alone or in combination. Data are expressed by setting the absorbance of the reduced MTT in control samples as 100%. Each histogram represent the results obtained from 4–8 independent experiments.

Confocal immunostaining experiments for phospho-histone H2A.X, a marker of activated DNA damage, indicate that a short treatment (1 h and 6 h) with 30 μg/ml climacostol caused a remarkable phosphorylation of H2A.X in B16-F10 cells (Fig. 2a). This further demonstrates that climacostol is a compound that rapidly forms adducts with DNA thus leading to DNA damage and cell death^19^. Induction of DNA damage has been shown to be an effective treatment of cancer and many used anti-tumoural drugs, as for instance platinum agents, act by inducing DNA damage^7^,^20^,^21^.

Cell viability was next assessed using the MTT assay in B16-F10 cells treated for 24 h with the chemotherapeutic compound cisplatin or climacostol at increasing concentrations (equals or below their EC_50_), or combinations of the two. As shown in Fig. 2b, cisplatin and climacostol did not show an additive action since melanoma cell viability in response to their combination was comparable to the viability achieved in the presence of the most effective single drug. The fact that cisplatin and climacostol did not display an additive interaction suggests that the sensitivity of melanoma cells in response to platinum chemotherapy remains unaltered in the presence of climacostol. This observation is in line with previous findings with drug combinations that include a DNA-targeting drug, including cisplatin, that have been frequently employed in therapeutic regimens with other chemotherapies^7^. Combinations of these drugs have shown response rates only marginally higher than those achieved with single drug administration and at the cost of increased systemic toxicity^7^.

### Climacostol-induced apoptosis in melanoma cells

**Figure 3:**
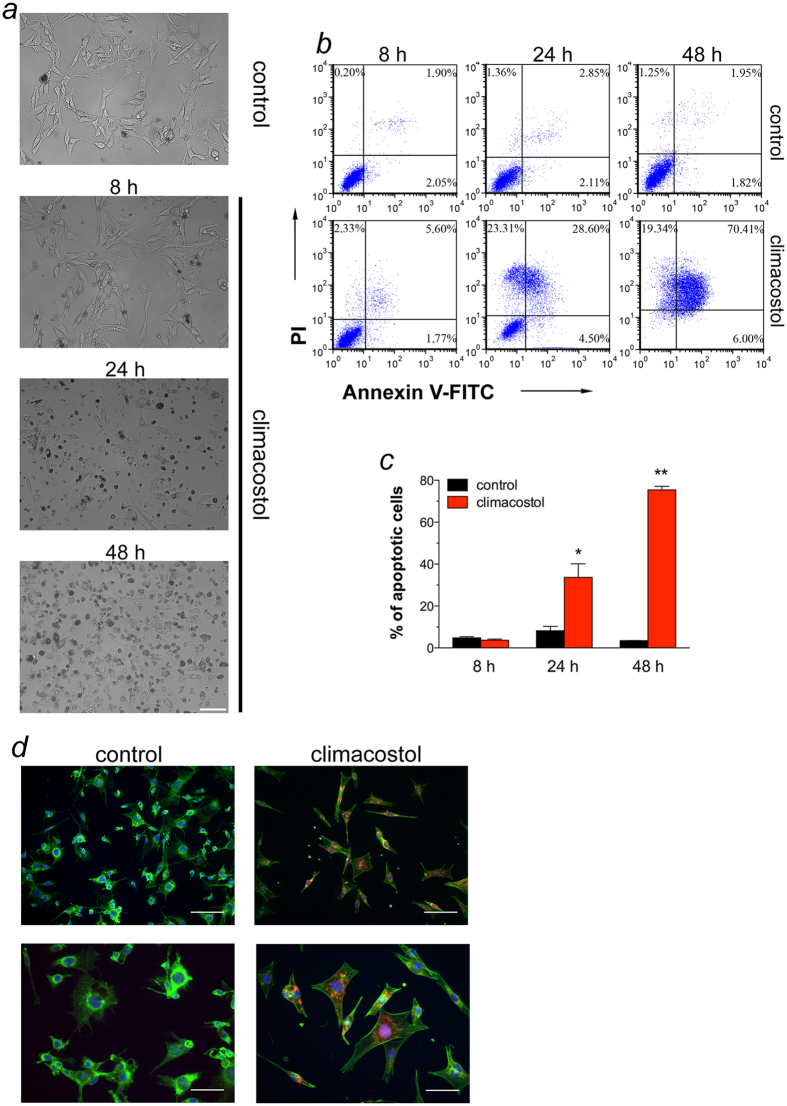
Apoptosis in melanoma cells treated with climacostol. (**a**–**c**) B16-F10 cells were cultured in the presence of 30 μg/ml climacostol or vehicle (control) for 8, 24 or 48 h. (**a**) Phase-contrast microscopy. The images are representative of 3 independent experiments. Scale bar: 50 μm. (**b**) Evaluation by flow cytometry of Annexin V/PI staining. Quadrants are drawn, and relative proportion of dying cells is indicated. The events shown in the lower left-hand quadrant are unlabeled cells. Images and data are representative of 3–7 independent experiments. (**c**) Percentage of apoptotic cells (Annexin V^+^/PI^−^ + Annexin V^+^/PI^+^ fraction; the Annexin V^−^/PI^+^ fraction, regarded as necrotic stage, was excluded) for experiments shown in (**b**). *p < 0.001 and **p < 0.0001 *vs* the respective control. (**d**) Immunofluorescence imaging of cleaved Caspase 3 (punctate red pattern) in B16-F10 cells cultured in the presence of 30 μg/ml climacostol or vehicle (control) for 24 h. DAPI (blue) and phalloidin (green) were used for nuclei and cytoskeleton detection, respectively. The images are representative of 3 independent experiments. Upper panels: 100 μm scale bar; lower panels: 50 μm scale bar.

Phase-contrast microscopy revealed that melanoma B16-F10 cells at 24 h, but not at 8 h, after exposure to 30 μg/ml climacostol became round, presented a shrunken cytoplasm and the formation of apoptotic bodies (Fig. 3a). Morphological modifications were also observed in the majority of cells exposed to climacostol for 48 h. In addition, flow cytometry analysis of phosphatidylserine exposure on the outer leaflet of the plasma membrane (a characteristic feature of apoptotic cell death) revealed that 30 μg/ml climacostol treatment for 24 and 48 h, but not 8 h, induced a significant increase in the Annexin V^+^/Propidium Iodide (PI)^−^ B16-F10 fraction (regarded as early apoptotic stage) and Annexin V^+^/PI^+^ B16-F10 fraction (regarded as late apoptotic/necrotic stage) (Fig. 3b,c). Overall apoptotic cells in the presence of climacostol at 24 and 48 h were ca. 35 and 75% of total cells, respectively. As expected, apoptosis in vehicle-treated cells (control) was almost undetectable. Accordingly, immunostaining with a fluorescently labelled antibody that binds specifically to cleaved (active) Caspase 3, an hallmark of apoptosis, revealed that B16-F10 cells expressed high levels of Caspase 3 activity at 24 h after 30 μg/ml climacostol treatment while no specific stain was observed in control cells (Fig. 3d). These results demonstrate the pro-apoptotic effects of climacostol in melanoma cells, in agreement with previous results^12^. It has been shown that climacostol can effectively bind both histone protected and naked DNA, promoting their cleavage in the presence of copper(II)^19^. Our data revealed that climacostol triggers a Caspase 3-dependent apoptotic programme in melanomas with enzymatic degradation of DNA. This enzymatic cleavage could reinforce the non-enzymatic one, thus playing an important role in explaining the preferential cytotoxicity of climacostol towards tumour cells that usually contain higher copper levels than normal cells^19^.

### Climacostol-induced activation of the mitochondrial-caspase-dependent apoptotic pathway in melanoma cells

**Figure 4:**
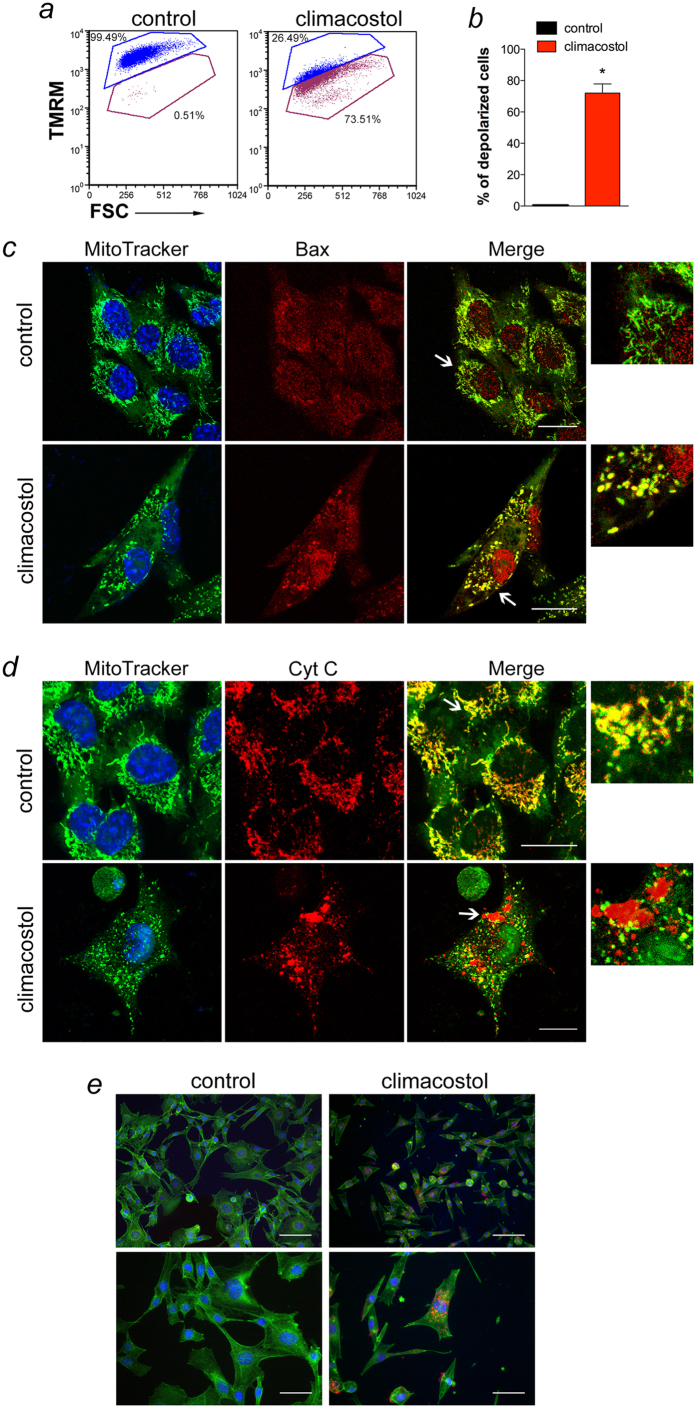
Climacostol activates mitochondrial-dependent apoptosis in melanoma cells. B16-F10 cells were cultured in the presence of 30 μg/ml climacostol or vehicle (control) for 24 h. (**a**) Evaluation by flow cytometry of mitochondrial potential, using TMRM staining. The percentage number of cells with intact mitochondrial potential (TMRM fluorescence in top gate) and percentage number of cells with reduced mitochondrial potential (depolarised cells; TMRM fluorescence in bottom gate). FSC: forward scatter. Images and data are representative of 3 independent experiments. (**b**) Percentage of depolarised cells for experiments shown in (**a**). *p < 0.0001 *vs* control. (**c**,**d**) Confocal microscopy for co-localization of Bax and Cytochrome c with mitochondria. Cells were stained for Bax or Cytochrome c (red) and the fluorescent mitochondrial dye MitoTracker Green. DAPI (blue) was used for nuclei detection. The images are representative of 3 independent experiments. Scale bar: 20 μm. Panels on the right represent enlarged image details marked by the white arrows. (**e**) Immunofluorescence imaging of cleaved Caspase 9 (punctate red pattern). DAPI (blue) and phalloidin (green) were used for nuclei and cytoskeleton detection, respectively. The images are representative of 3 independent experiments. Upper panels: 100 μm scale bar; lower panels: 50 μm scale bar.

In human pro-myelocytic leukemia, but not in squamous carcinoma cells, climacostol was suggested to induce cell death by activating the intrinsic apoptotic programme^12^. We now demonstrate and further characterise this mechanism in melanoma. In particular, by using flow cytometry analysis of tetramethylrhodamine methyl ester (TMRM) staining we found that about 70% of B16-F10 cells treated for 24 h with 30 μg/ml climacostol displayed depolarised mitochondria (Fig. 4a,b). The reduction of the mitochondrial membrane potential indicated that the integrity of mitochondrial structure was disrupted after climacostol treatment. The dissipation of mitochondrial membrane potential with the formation of permeability transition pore is recognised as an early stage of apoptosis, where the translocation of Bax to the mitochondria and Cytochrome c release from the mitochondria into the cytoplasm signal the initiation of apoptosis. Cytochrome c, in turn, activates Caspase 9-dependent apoptotic disassembly and Caspase 9-dependent proteolytic cleavage of Caspase 3. In the present study, confocal immunofluorescence staining was utilised to identify the subcellular location of Bax and Cytochrome c in melanoma cells. As shown in Fig. 4c, Bax gave a coarse staining pattern in vehicle-treated (control) cells, which did not co-localise with mitochondria as determined using MitoTracker Green. However, 30 μg/ml climacostol treatment (24 h) resulted in a punctate Bax staining pattern with evidence of mitochondrial clustering due to its extensive overlapping with MitoTracker Green. The mitochondrial clustering of Bax was associated with an alteration in the Cytochrome c staining pattern from mitochondrial (co-localization with MitoTracker Green), to a more cytosolic distribution (presence of many clusters which did not overlap with MitoTracker Green), indicating a release of Cytochrome c from the mitochondria (Fig. 4d). In addition, exposure of B16-F10 cells to climacostol (24 h, 30 μg/ml) induced the expression of high levels of cleaved (active) Caspase 9 while no specific immunofluorescence stain was observed in control cells (Fig. 4e). Considering this mechanistic insight in melanoma cells, our data are consistent with the induction of an intrinsic apoptotic pathway, characterised by the central role of the mitochondria dysfunction in the initiation of the pro-apoptotic effects of climacostol. Of interest, melanoma drug resistance is often attributed to defect in the intrinsic apoptosis pathway. Targeting regulators of mitochondria-mediated apoptosis is thus considered a promising approach to sensitising melanomas to treatment^22^.

### Climacostol induces p53-dependent apoptosis in melanoma cells

**Figure 5:**
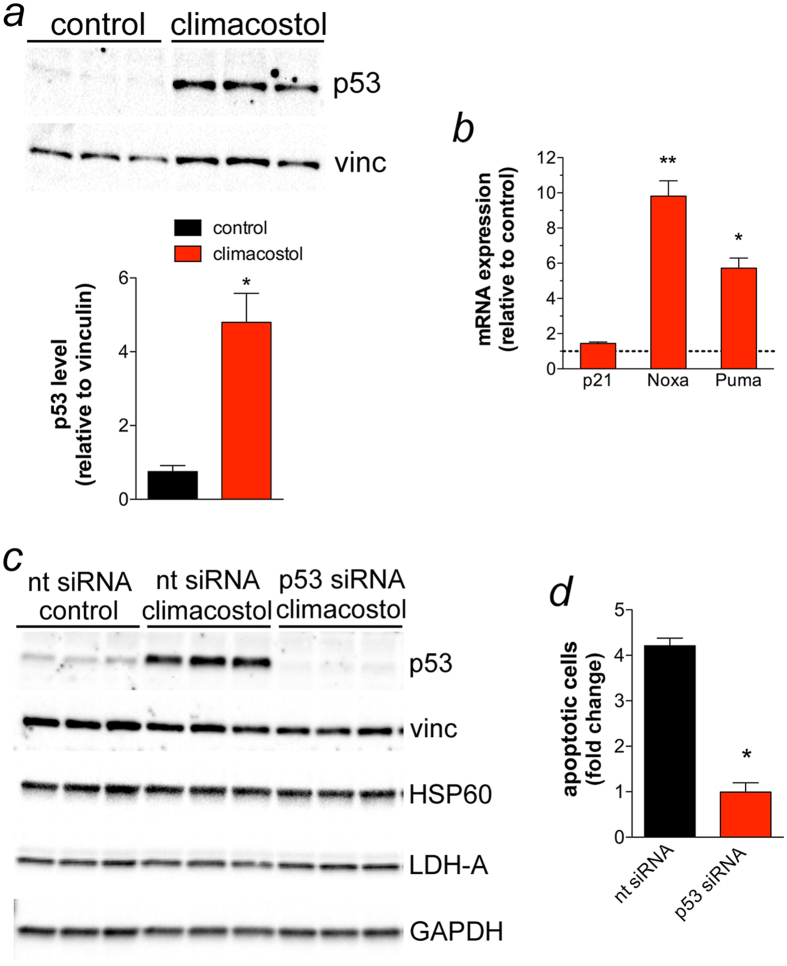
Pro-apoptotic effects of climacostol in melanoma cells depend on p53 activity. (**a**,**b**) B16-F10 cells were cultured in the presence of 30 μg/ml climacostol or vehicle (control) for 24 h. (**a**) Western blot analysis of p53 expression. Vinculin was used as the internal standard; Lower panel: densitometry analysis of p53 expression (n = 3) *p < 0.001 *vs* control. (**b**) mRNA levels of p53 target genes p21, Noxa and Puma, as measured by real-time PCR. Each histogram represent the results obtained from 3 independent experiments. Data are expressed as the fold change over control (set as 1). *p < 0.001 and **p < 0.0001 *vs* the respective control. (**c**,**d**) B16-F10 cells were transiently transfected for 48 h with a p53-specifc (p53 siRNA) and a non-targeting siRNA (nt siRNA). Cells were then treated for 24 h with 30 μg/ml climacostol or vehicle (control). (**c**) Western blot analysis of p53 expression. Vinculin, HSP60, LDH-A, and GAPDH were used as internal standards and to test possible off-target effects of siRNA transfection (n = 3). (**d**) Evaluation by flow cytometry of Annexin V/PI staining (Annexin V^+^/PI^−^ + Annexin V^+^/PI^+^ fraction; the Annexin V^−^/PI^+^ fraction, regarded as necrotic stage, was excluded). Data are expressed as fold change over the respective control. Each histogram represent the results obtained from 3 independent experiments. *p < 0.001 *vs* nt siRNA.

Numerous genotoxic stresses interfere with the integrity of the genome; hence, cells maintain various mechanisms to avoid genetic alterations that would otherwise cause cell transformation. The tumour suppressor p53 is the most prominent factor for the maintenance of genome integrity in response to DNA damage and plays a key role in determining the fate of cells^23^. In particular, p53 and the products of its activity can either promote apoptosis (i.e. Noxa, Puma), or induce cell cycle arrest and DNA damage repair (i.e. p21) in different systems, including melanomas^24^,^25^,^26^,^27^,^28^,^29^. Of interest, p53 stimulates a wide network of signals that may also act through the intrinsic mitochondrial apoptotic pathway. In particular, it has been shown that p53, Noxa and Puma induction in melanomas promote mitochondrial translocation and multimerisation of Bax thus activating Cytochrome c-dependent caspases^24^,^25^,^26^,^27^,^28^,^29^. As shown Fig. 5a, exposure of B16-F10 cells for 24 h to 30 μg/ml climacostol resulted in a significant increase of p53 protein levels when compared to vehicle-treated (control) cells. In addition, the mRNA expression of Noxa and Puma, but not p21, was found to be significantly up-regulated by climacostol (Fig. 5b).

We then determined whether the pro-apoptotic effect of climacostol involved p53 signalling. To this end, B16-F10 cells were transiently transfected for 48 h with a p53-specific or a non-targeting siRNA, followed by climacostol treatment (24 h, 30 μg/ml). Vehicle was also used as control. As shown in Fig. 5c, climacostol markedly increased p53 levels in cells transfected with non-targeting siRNA while its effect was completely inhibited after p53 siRNA transfection. The analysis of different unrelated proteins did not show off-target effects of siRNA treatments. Of interest, the analysis of Annexin V^+^/Propidium Iodide (PI)^−^ and Annexin V^+^/PI^+^ B16-F10 fractions (apoptotic cells) revealed that p53 silencing abolished the apoptotic effects of climacostol (Fig. 5d) thus indicating that the mechanism by which climacostol induces apoptosis in B16-F10 melanomas is mainly, if not completely, dependent on p53 activity. The effects of climacostol are in line with the role of anti-tumour agents, including cisplatin, leading to melanoma cell death with changes in the expression of p53 and its signals^20^,^21^.

### Climacostol inhibits melanoma allografts growth *in vivo*, increases animal survival and apoptosis, and induces p53 network

**Figure 6:**
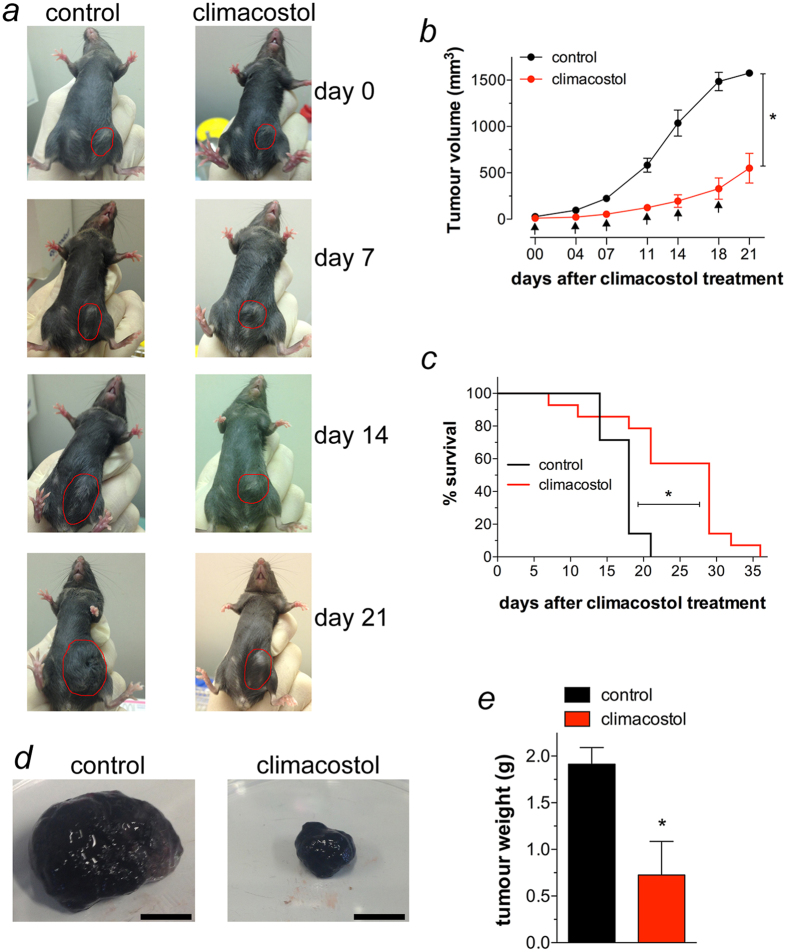
*In vivo* anti-tumour properties of climacostol in mice bearing melanoma allografts. When the B16-F10 syngeneic implantation was established animals were intra-tumour injected with vehicle (control) or climacostol at 600 μg/ml every 3–4 days for 3 weeks. (**a**) Typical photographs taken at different time-points and depicting the growth of subcutaneous melanomas, as indicated by red circles. (**b**) Tumour growth monitored by means of external caliper measurements and volume calculation. Arrows indicate the day of climacostol treatment. (**c**) Percentage survival analysed by Kaplan-Meier curve. Images and data points in (**a–c**) represent the results obtained from 7–15 animals per experimental group. (**d**) Typical photographs of subcutaneous melanoma allografts excised from mice at day 16 of treatment (from day 0 - every 3–4 days) with vehicle (control) or climacostol (600 μg/ml). Scale bar: 5 mm. (**e**) Weight of the excised tumours. Images and data in (**d**,**e**) represent the results obtained from 3 animals per experimental group. *p < 0.001 *vs* the respective control.

The cytotoxic effect of most of the marketed drugs used against melanomas typically occurs through (intrinsic) programed cell death^3^,^22^. In addition, local administration is a potential approach for delivering high doses of drugs at the target tumour site while minimising systemic exposure. Indeed, conventional chemo-and immunotherapy is severely limited because of its systemic toxicity and inefficiency. Accordingly, several strategies of regional delivery have been used to optimise the drug effects to the melanoma site *in vivo*^30^,^31^, as for instance intra-tumour administration of cisplatin, and with minimal adverse effects^32^. In light of this and the present definition of the *in vitro* cytotoxic activity against melanoma cells, the efficacy of climacostol was tested *in vivo*. To this end B16-F10 cells were injected subcutaneously in mice; when the syngeneic implantation was established, mice were intra-tumour injected with vehicle (control) or climacostol at 600 μg/ml, in total volume of 100 μl, every 3–4 days for 3 weeks. As shown in Fig. 6a, local administration of climacostol decreased tumour volumes throughout the entire study period. In particular, as shown in the melanoma volume analysis of Fig. 6b, tumour growth rate increased steadily for vehicle-treated animals (control) while we observed a significant and persistent inhibition of tumour load in the case of transplants treated with climacostol. In agreement with these results, the Kaplan-Meier analysis of Fig. 6c revealed that climacostol significantly improved the survival of B16-F10 melanoma-injected mice (median of survival: control = 18 days, climacostol-treated group = 29 days).

**Figure 7:**
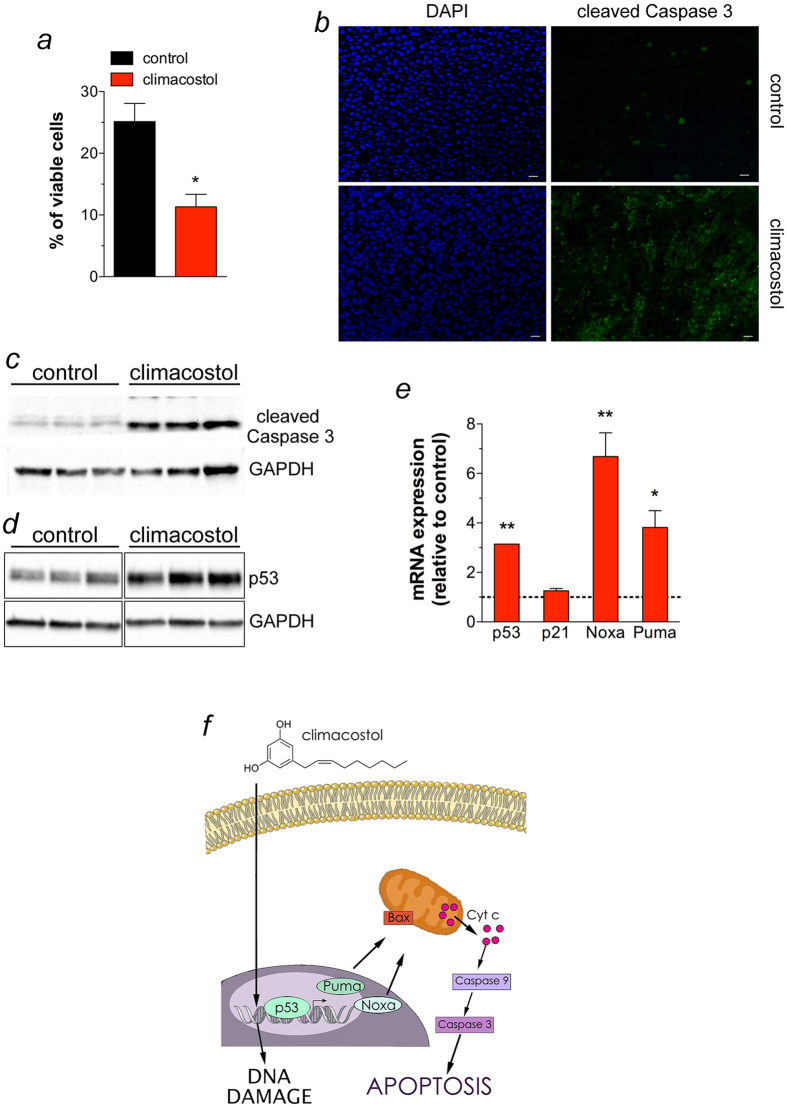
*In vivo* pro-apoptotic properties of climacostol in mice bearing melanoma allografts. Subcutaneous B16-F10 melanoma allografts were excised from mice at day 16 of treatment (from day 0 - every 3–4 days) with vehicle (control) or climacostol (600 μg/ml). (**a**) Percentage of viable cells inside the tumours as evaluated by Trypan Blue staining. (**b**) Representative immunofluorescence imaging of cleaved Caspase 3. DAPI (blue) was used for nuclei detection. Scale bar: 20 μm. (**c**,**d**) Western blot analysis of cleaved Caspase 3 and p53 expression, respectively. GAPDH was used as the internal standard. (**e**) mRNA levels of p53 and its target genes p21, Noxa and Puma, as measured by real-time PCR. Data are expressed as the fold change over control (set as 1). Images and data represent the results obtained from 3 animals per experimental group. *p < 0.001 and **p < 0.0001 *vs* the respective control. (**f**) Schematic picture depicting cell death mechanisms of climacostol in melanomas.

In separate experiments, B16-F10 tumours were removed at day 16 of treatment with local administration of climacostol at 600 μg/ml. As shown in Fig. 6d,e, climacostol significantly decreased melanoma weight by ca. 60% when compared to vehicle-treated group (control). Consistently, climacostol exposure induced a remarkable reduction of viable cells inside the tumour (ca. 55% reduction *vs* control), as assessed by analysis of Trypan Blue staining (Fig. 7a). In addition, immunostaining experiments of the cleaved Caspase 3 revealed an high amount of clustered apoptotic cells in tumours treated with climacostol while the stain for the cleaved Caspase 3 was scarce in control tumours (Fig. 7b). These results were confirmed by Western blot experiments since climacostol-treated melanomas showed an increase of active Caspase 3 when compared to control (Fig. 7c). In agreement with the *in vitro* results, also the expression of p53 was enhanced in melanomas treated with climacostol (Fig. 7d). Accordingly, we also found a significant increase of p53, Noxa and Puma mRNAs in the climacostol-treated group, while mRNA expression of p21 was not affected (Fig. 7e).

Taken together, our *in vitro* and *in vivo* results indicate that climacostol exerts an effective inhibitory action on B16-F10 mouse melanoma progression thus increasing animal survival. In particular, climacostol triggers the death process of tumour cells as a result of an early DNA binding and damage; this phenomenon is then responsible of the induction of apoptosis. Of notice, the signalling events responsible of the climacostol-induced pro-apoptotic effects rely on the up-regulation of p53 network that, in turn, activates the intrinsic programed cell death pathway (Fig. 7f).

## Conclusions

Cancer cells develop ways to evade apoptosis, or exhibit defective apoptosis mechanisms, thus allowing uncontrollable cell development. In this respect, melanoma is characterised by an imbalance towards too little apoptosis, or too much cell proliferation and survival in the epidermis^33^. Melanoma has also a close relationship with the immune system, with tumour infiltration by immune cells often indicating their attempt to eliminate the tumour. Noteworthy, melanoma is a disease characterised by cutaneous, subcutaneous and nodal metastases, which has led to study various intra-lesion therapies^30^,^31^. Indeed, the ease of drug delivery of localised treatments makes them attractive particularly in melanomas^30^,^31^.

Once an untreatable disease, now melanoma can be targeted through a variety of recently developed drugs. While these drugs have ameliorated significantly the overall time of survival still a definite cure for melanoma is lacking. A striking feature of melanoma is also the development of resistance to treatment over time. A possible strategy to face these issues is to combine the new targeted drugs and immunotherapies with compounds that overcome the ability of melanoma cells to respond to damage and eventually modulate the apoptotic programme. Many compounds of natural origin with diverse chemical structure and therapeutic potential in melanoma are cytotoxic and able to induce apoptosis^8^,^9^. This study now shows that one of them, *i.e.* climacostol, is also a powerful and efficacious anti-cancer agent targeting the intrinsic apoptotic programme linked to p53 system, which may be used *in vivo*, for instance, in intra-lesion therapy approaches. Although the therapeutic potential of climacostol by systemic administration is still unknown, and deserves to be investigated, our results provide a rationale to consider climacostol for the design of cytotoxic and pro-apoptotic new drugs for melanoma therapy.

## Materials and Methods

### Climacostol and chemicals

Chemically synthesised climacostol (C_15_H_22_O_2_) was obtained as previously described^13^ as a substantially pure compound, with a purity major than 99% determined by spectral analysis^34^, avoiding contamination with the undesired (*E*)-diastereomer present in the natural toxin purified from cultures of *C. virens*. Synthetic climacostol was then dissolved in absolute ethanol at 10 mg/ml stock, and stored in the dark at −20 °C until use. Climacostol dilutions in *in vitro* experiments were prepared with the appropriate culture medium. The stock solution of climacostol was diluted in phosphate buffered saline for *in vivo* injections.

Foetal bovine serum, phosphate buffered saline, bovine serum albumin (BSA), Iscove’s modified Dulbecco’s medium, and other chemicals used in cell cultures were purchased from Euroclone (Pero, Italy). Cisplatin (Cisplatino Teva) was from Teva Pharma Italia (Milano, Italy). Annexin V-Fluorescein Isothiocianate (FITC), dispase, DNase I, and fluorescein phalloidin were obtained from Life Technologies (Monza, Italy). PI and Trypan Blue were purchased from eBioscience (San Diego, CA, USA) and Bio-Rad (Hercules, CA, USA), respectively. Primer pairs were obtained from Eurofins Genomics (Milano, Italy). All other reagents were purchased from Sigma-Aldrich (Saint Louis, MO, USA).

### Cell cultures

Hela, PC12, AtT-20, NIH/3T3, B16-F10^25^,^26^,^27^,^28^,^29^,^30^,^31^,^32^, P3X and TM3 cells (American Type Culture Collection) were cultured at 37 °C, 5% CO_2_ in an humidified atmosphere. Cells were maintained in the presence of 10% foetal bovine serum-containing medium and in logarithmic growth phase (routine passages every 3 days).

### LDH activity and MTT assay

The cytotoxic potential of climacostol was determined analysing LDH activity (CytoScan LDH cytotoxicity assay, G-Biosciences, Saint Louis, MO, USA) in the supernatant of TM3 cells, as detailed in Buonanno and coll^12^. Using published protocols^16^,^35^,^36^,^37^,^38^,^39^,^40^, cell viability of P3X, PC12, AtT-20, 3T3, and B16-F10 cells was evaluated by MTT analysis. MTT absorbance was quantified spectrophotometrically using a Glomax Multi Detection System microplate reader (Promega, Milano, Italy).

### Proliferation assay

B16-F10 cell proliferation was assessed by measuring the serial halving of cell fluorescence intensity via flow cytometry^39^. The CytoTrack Cell Proliferation Assays (CytoTrack Green; Bio-Rad, Hercules, CA, USA) was used, according to the manufacturer’s protocol. CytoTrack Assay dye is evenly distributed in the cytoplasm and stoichiometrically distributed to daughter cells during cell division. The fluorescence was analysed by Gallios Flow Cytometer (Beckman-Coulter, Brea, CA, USA) and the software FCS Express 4 (De Novo System, Portland, OR, USA). The proliferation index, defined as the average number of cells that an initial cell became, was calculated using FCS Express software.

### Annexin V-FITC/PI staining and mitochondrial membrane potential

B16-F10 cells were incubated with 5 μg/ml Annexin V-FITC to assess the phosphatidylserine exposure on the outer leaflet of the plasma membrane, and 5 μg/ml PI (DNA-binding probe) to exclude necrotic cells in binding buffer (10 mM HEPES, 140 mM NaCl, 2.5 mM CaCl_2_)^39^,^41^,^42^,^43^. Mitochondrial potential was measured by cell staining with 500 nM of sensitive fluorescent dye, TMRM (Sigma-Aldrich, Saint Louis, MO, USA), according to the manufacturer’s protocol. Cell staining was acquired by flow cytometry.

### Microscopy analysis

B16-F10 cells cultured in 120-mm coverslips were visualised by phase-contrast microscopy. Mitochondria were stained using MitoTracker Green FM (ThermoFisher Scientific, Waltham, MA, USA), according to the manufacturer’s protocol. As previously published^38^, cells were fixed in 4% paraformaldehyde and stained with the rabbit polyclonal anti-cleaved Caspase 3, anti-cleaved Caspase 9, anti-Bax, or the mouse monoclonal anti-Cytochrome c (Cell Signaling Technology, Danvers, MA, USA) and anti-phospho-histone H2A.X (ser139) (Merck Millipore, Darmstadt, Germany) primary antibodies. For fluorescence detection, cells were also stained with the appropriate Alexa Fluor secondary antibodies (Life Technologies, Monza, Italy). Coverslips were mounted on glass slides in a ProLong Gold Antifade Mountant (Life Technologies, Monza, Italy), stained with DAPI (nuclei detection) and/or fluorescein phalloidin (cytoskeleton detection), and analysed using a DMI4000 B automated inverted microscope equipped with a DCF310 digital camera (Leica Microsystems, Wetzlar, Germany). Confocal imaging was performed with a Leica TCS SP5 AOBS microscope system. Image acquisitions were controlled by the Leica LAS AF software.

For melanoma immunostaining^18^, *in vivo* resected B16-F10 tumours were immersion-fixed in 4% paraformaldehyde in 0.1 M phosphate buffer (pH 7.4) for 3 h. The fixed tissue were transferred to 25% sucrose in phosphate buffer. Tumour sections were cut at 10 μm with a cryostat, mounted onto positive charged slides and stored at −20 °C. For Caspase 3 staining, sections were pre-incubated for 15 min with 5% BSA and 10% of normal goat serum (Life Technologies, Monza, Italy) in 0.1 M phosphate buffer (pH 7.4) containing 0.5% Triton X-100. Pre-treated sections were incubated overnight with the rabbit polyclonal anti-cleaved Caspase 3 primary antibody (Cell Signaling Technology, Danvers, MA, USA). For fluorescence detection, sections were stained with the appropriate Alexa Fluor secondary antibody (Life Technologies, Monza, Italy) and cover slipped with a phosphate buffer-glycerin mixture containing DAPI. Incubation in secondary antibody alone was performed as negative control. Images were acquired by a Zeiss Axioskop 2 plus microscope equipped with the Axiocam MRC photocamera and the Axiovision software (Carl Zeiss, Oberkochen, Germany).

### Western blotting

Using published protocols^16^,^38^,^39^,^44^,^45^, B16-F10 cells and *in vivo* resected B16-F10 tumours were homogenized in RIPA lysis buffer, supplemented with a cocktail of protease and phosphatase inhibitors (cOmplete and PhosSTOP; Roche Diagnostics, Milano, Italy). Equal amounts of proteins were separated by 4–20% SDS-polyacrylamide gel electrophoresis (Criterion TGX Stain-free precast gels and Criterion Cell system; Bio-Rad, Hercules, CA, USA) and transferred onto nitrocellulose membrane using a Bio-Rad Trans-Blot Turbo System. The membranes were probed using the mouse monoclonal anti-p53 and the rabbit polyclonal anti-cleaved Caspase 3 primary antibodies (Cell Signaling Technology, Danvers, MA, USA). After the incubation with the appropriate horseradish-peroxidase-conjugated secondary antibody (Cell Signaling Technology, Danvers, MA, USA), bands were visualised using the Clarity Western ECL substrate with a ChemiDoc MP imaging system (Bio-Rad, Hercules, CA, USA). To monitor for potential artefacts in loading and transfer among samples in different lanes, the blots were routinely treated with the Restore Western Blot Stripping Buffer (ThermoFisher Scientific, Waltham, MA, USA) and re-probed with the mouse monoclonal anti-vinculin (Sigma-Aldrich, Saint Louis, MO, USA), goat polyclonal anti-HSP60 or anti-LDH-A, and rabbit polyclonal anti-GAPDH, (Santa Cruz Biotechnology, Dallas, TX, USA) primary antibodies. When appropriated, bands were quantified for densitometry using the Bio-Rad Image Lab software.

### Real-time PCR

The analysis of mRNA expression was performed as previously described^16^,^38^,^44^,^46^,^47^. Briefly, total RNA from in vitro B16-F10 cells and *in vivo* resected B16-F10 tumours was extracted with the High Pure RNA Isolation Kit and the High Pure RNA Tissue Kit, respectively (Roche Applied Science, Mannheim, Germany), according to the manufacturer’s protocol. First-strand cDNA was generated from 1 μg of total RNA using iScript Reverse Transcription Supermix (Bio-Rad, Hercules, CA, USA). A set of primer pairs was designed to hybridize to unique regions of the appropriate gene sequence (Supplementary Table S1). PCR was performed using SsoAdvanced Universal SYBR Green Supermix and the CFX96 Touch Real-Time PCR Detection System (Bio-Rad). The fold change was determined relative to the control after normalizing to Rpl32 (internal standard) through the use of the formula 2^−ΔΔCT^.

### Gene silencing of p53

According to the manufacturer’s protocol, iBONI siRNA Pool (a mix of 3 RNA duplex - small interfering RNAs) (Riboxx, Radebeul, Germany) targeting mouse p53 (gene ID 22059; Supplementary Table S2) was mixed to Lipofectamine RNAiMax transfection reagent (Life Technologies, Monza, Italy). iBONI siRNA Pool negative control (non-targeting siRNA; Supplementary Table S2) (Riboxx, Radebeul, Germany) was also used. The mix was added to cultured B16-F10 cells at a siRNA concentration of 50 nM. At 48 h after transfection, climacostol (24 h, 30 μg/ml) was then added before Western blotting and flow cytometry analyses.

### Animals

Female C57BL/6 mice (8–10 weeks old) were purchased from Charles River Laboratories (Calco, Italy), housed in a regulated environment (23 ± 1 °C, 50 ± 5% humidity) with a 12 h light/dark cycle (lights on at 08.00 a.m.), and provided with food and water ad libitum. All studies were conducted in accordance with the Italian law on animal care N° 116/1992 and the European Communities Council Directive EEC/609/86. The experimental protocol (N° 04/11, Department of Biomedical and Clinical Sciences “Luigi Sacco”, Università di Milano) were approved by the Ethics Committee of the Università degli Studi di Milano. All efforts were made to reduce both animal suffering and the number of animals used.

### Animal handling and allograft tumour model

Using published protocols^16^,^17^,^42^, mice (weighing 16–18 g) received subcutaneous injections of 5 × 10^4^ cells B16-F10 in the lower-right flank (seven animals per group at minimum). When the syngeneic implantation was established (usually 10 days after tumour cells inoculation) and the tumour was palpable (volume range between 15–30 mm^3^), mice with almost similar size of tumours were randomly assigned to one of the two experimental groups. In particular, transplanted mice received 100 μl intra-tumour injections of vehicle or climacostol (600 μg/ml, equivalent to ca. 3.5 mg/kg dose, respectively) every 3–4 days for 3 weeks (end at day 18). Mice were then photographed and tumour growth was monitored twice a week by means of external calliper measurements and volume calculation (length × width^2^/2) until mice reached IACUC euthanasia criteria, as for instance clinical signs of tumour or when tumour size exceeded 10% of body weight (tumour volume ca. 1500 mm^3^)^16^,^17^,^42^. Studies were repeated once to determine the repeatability of results. The mice were also observed to determine the duration of survival for each group using the Kaplan-Meier estimator (median survival).

Different groups of mice were also sacrificed at day 16. Tumours were removed, photographed and weighted before immunostaining, Western blot, and real-time PCR experiments as detailed above. A single cell suspension from tumours was also obtained by collagenase IV (0.2 mg/ml), dispase (2 mg/ml) and DNase I (0.1 mg/ml) treatment in Iscove’s modified Dulbecco’s medium^16^,^17^. Cell viability was measured after 0.2% Trypan Blue staining by counting trypan blue-excluding cells with a TC20 Automated Cell Counter (Bio-Rad, Hercules, CA, USA).

### Statistics

EC_50_ (the concentration producing half the maximum effect) and E_max_ concentration (producing the maximum effect) was determined by non-linear regression curve analysis of the concentration-effect responses. Differences in potency values among concentration-response curves were calculated with the F test. Kaplan-Meyer data were analysed with the multiple comparison survival curve method using the Log-rank (Mantel-Cox) test. Upon verification of normal distribution (ShapiroWilks test), statistical significance of raw data between the groups in each experiment was evaluated using unpaired Student’s t-test (single comparisons) or one-way ANOVA followed by the Newman-Keuls post-test (multiple comparisons). Tumour growth was analysed using two-way ANOVA, followed by the Bonferroni post-test. When indicated, data belonging from different experiments were represented and averaged in the same graph. The GraphPad Prism software package (Graph Software, San Diego, CA, USA) was used. The results were expressed as means ± SEM of the indicated n values.

## Additional Information

**How to cite this article**: Perrotta, C. *et al.* Climacostol reduces tumour progression in a mouse model of melanoma via the p53-dependent intrinsic apoptotic programme. *Sci. Rep.* **6**, 27281; doi: 10.1038/srep27281 (2016).

## Supplementary Material

Supplementary Information

## Figures and Tables

**Table 1:** Parameters of climacostol-induced inhibition of cell viability: tumourigenic and non-tumourigenic cell lines.

Cell line	Origin	Species	EC_50_	Assay	Source
HeLa	cervix carcinoma	human	0.605	MTT	*
A431	squamous carcinoma	human	~2.0	MTT/LDH	12
HL60	pro-myelocytic leukemia	human	~2.0	MTT/LDH	12
PC-3	prostatic adenocarcinoma	human	2.685	MTT	13
T98G	glioblastoma	human	3.545	MTT	13
U87MG	glioblastoma	human	4.627	MTT	13
P3X	myeloma	mouse	5.665	MTT	*
PC12	pheochromocytoma	rat	5.702	MTT	*
atT-20	pituitary adenoma	mouse	6.232	MTT	*
TM3	Leydig cells	mouse	11.30	LDH	*
NIH/3T3	fibroblasts	mouse	12.16	MTT	*
EA.hy926	endothelial cells	human	>50	MTT/LDH	12,13

EC_50_ (μg/ml) = concentration required to produce 50% of the effects. MTT or LDH assays were performed treating cells for 24 h in the absence (vehicle) or in the presence of increasing concentrations of climacostol.

^*^Present paper, *n* = 3.

## References

[b1] Jemal A., Siegel R., Xu J. & Ward E. Cancer statistics. CA: a cancer journal for clinicians 60, 277–300 (2010). 20610543 10.3322/caac.20073

[b2] Ferlay J., Parkin D. M. & Steliarova-Foucher E. Estimates of cancer incidence and mortality in Europe in 2008. Eur J Cancer 46, 765–781 (2010). 20116997 10.1016/j.ejca.2009.12.014

[b3] Megahed A. I. & Koon H. B. What is the role of chemotherapy in the treatment of melanoma? Current treatment options in oncology 15, 321–335 (2014). 24599525 10.1007/s11864-014-0277-5

[b4] Michielin O. & Hoeller C. Gaining momentum: New options and opportunities for the treatment of advanced melanoma. Cancer Treat Rev 41, 660–670 (2015). 26096079 10.1016/j.ctrv.2015.05.012

[b5] Hao M. *et al.* Advances in targeted therapy for unresectable melanoma: new drugs and combinations. Cancer Lett 359, 1–8 (2015). 25578781 10.1016/j.canlet.2014.12.050

[b6] Marzuka A., Huang L., Theodosakis N. & Bosenberg M. Melanoma Treatments: Advances and Mechanisms. J Cell Physiol 230, 2626–2633 (2015). 25899612 10.1002/jcp.25019

[b7] Nikolaou V. A., Stratigos A. J., Flaherty K. T. & Tsao H. Melanoma: new insights and new therapies. J Invest Dermatol 132, 854–863 (2012). 22217739 10.1038/jid.2011.421 PMC3759013

[b8] Chinembiri T. N., du Plessis L. H., Gerber M., Hamman J. H. & du Plessis J. Review of natural compounds for potential skin cancer treatment. Molecules 19, 11679–11721 (2014). 25102117 10.3390/molecules190811679 PMC6271439

[b9] AlQathama A. & Prieto J. M. Natural products with therapeutic potential in melanoma metastasis. Nat Prod Rep 32, 1170–1182 (2015). 26018751 10.1039/c4np00130c

[b10] Miyake A., Buonanno F., Saltalamacchia P., Masaki M. E. & Iio H. Chemical defence by means of extrusive cortical granules in the heterotrich ciliate Climacostomum virens. Eur J Protistol 39, 25–36 (2003).

[b11] Buonanno F., Harumoto T. & Ortenzi C. The Defensive Function of Trichocysts in Paramecium tetraurelia Against Metazoan Predators Compared with the Chemical Defense of Two Species of Toxin-containing Ciliates. Zool. Sci. 30, 255–261 (2013). 23537235 10.2108/zsj.30.255

[b12] Buonanno F. *et al.* The protozoan toxin climacostol inhibits growth and induces apoptosis of human tumor cell lines. Chem Biol Interact 176, 151–164 (2008). 18723007 10.1016/j.cbi.2008.07.007

[b13] Fiorini D. *et al.* A Straightforward Diastereoselective Synthesis and Evaluation of Climacostol, A Natural Product with Anticancer Activities. Synthesis (tuttg) 9, 1550–1556 (2010).

[b14] Petrelli D., Buonanno F., Vitali L. A. & Ortenzi C. Antimicrobial activity of the protozoan toxin climacostol and its derivatives. Biologia 67, 525–529 (2012).

[b15] Buonanno F. & Ortenzi C. The protozoan toxin climacostol and its derivatives: Cytotoxicity studies on 10 species of free-living ciliates. Biologia 65, 675–680 (2010).

[b16] Bizzozero L. *et al.* Acid sphingomyelinase determines melanoma progression and metastatic behaviour via the microphtalmia-associated transcription factor signalling pathway. Cell Death Differ 21, 507–520 (2014). 24317198 10.1038/cdd.2013.173 PMC3950316

[b17] Assi E. *et al.* Modulation of Acid Sphingomyelinase in Melanoma Reprogrammes the Tumour Immune Microenvironment. Mediators Inflamm 2015, 370482 (2015). 26101462 10.1155/2015/370482 PMC4460251

[b18] Cervia D. *et al.* Essential role for acid sphingomyelinase-inhibited autophagy in melanoma response to cisplatin. Oncotarget 7, 24995–25009 (2016). 10.18632/oncotarget.8735 PMC5041885 27107419

[b19] Quassinti L. *et al.* DNA binding and oxidative DNA damage induced by climacostol-copper(II) complexes: implications for anticancer properties. Chem Biol Interact 206, 109–116 (2013). 23994247 10.1016/j.cbi.2013.08.007

[b20] Siddik Z. H. Cisplatin: mode of cytotoxic action and molecular basis of resistance. Oncogene 22, 7265–7279 (2003). 14576837 10.1038/sj.onc.1206933

[b21] Basu A. & Krishnamurthy S. Cellular responses to Cisplatin-induced DNA damage. Journal of nucleic acids 2010, 201367 (2010). 20811617 10.4061/2010/201367 PMC2929606

[b22] Mohana-Kumaran N., Hill D. S., Allen J. D. & Haass N. K. Targeting the intrinsic apoptosis pathway as a strategy for melanoma therapy. Pigment Cell Melanoma Res 27, 525–539 (2014). 24655414 10.1111/pcmr.12242

[b23] Yoshida K. & Miki Y. The cell death machinery governed by the p53 tumor suppressor in response to DNA damage. Cancer Sci 101, 831–835 (2010). 20132225 10.1111/j.1349-7006.2009.01488.x PMC11158978

[b24] Dashzeveg N. & Yoshida K. Cell death decision by p53 via control of the mitochondrial membrane. Cancer Lett 367, 108–112 (2015). 26231733 10.1016/j.canlet.2015.07.019

[b25] Qin J. Z., Xin H., Sitailo L. A., Denning M. F. & Nickoloff B. J. Enhanced killing of melanoma cells by simultaneously targeting Mcl-1 and NOXA. Cancer Res 66, 9636–9645 (2006). 17018621 10.1158/0008-5472.CAN-06-0747

[b26] Yu K. S. *et al.* The protease inhibitor, elafin, induces p53-dependent apoptosis in human melanoma cells. Int J Cancer 127, 1308–1320 (2010). 20020498 10.1002/ijc.25125

[b27] Hussein M. R., Haemel A. K. & Wood G. S. Apoptosis and melanoma: molecular mechanisms. J Pathol 199, 275–288 (2003). 12579529 10.1002/path.1300

[b28] Yu F., Watts R. N., Zhang X. D., Borrow J. M. & Hersey P. Involvement of BH3-only proapoptotic proteins in mitochondrial-dependent Phenoxodiol-induced apoptosis of human melanoma cells. Anticancer Drugs 17, 1151–1161 (2006). 17075314 10.1097/01.cad.0000231484.17063.9a

[b29] Nandy A. *et al.* Gold (I) N-heterocyclic carbene complex inhibits mouse melanoma growth by p53 upregulation. Mol Cancer 13, 57 (2014). 24625085 10.1186/1476-4598-13-57 PMC4007776

[b30] Sloot S., Rashid O. M. & Zager J. S. Intralesional therapy for metastatic melanoma. Expert Opin Pharmacother 15, 2629–2639 (2014). 25381015 10.1517/14656566.2014.967682

[b31] Agarwala S. S. Intralesional therapy for advanced melanoma: promise and limitation. Curr Opin Oncol 27, 151–156 (2015). 25629369 10.1097/CCO.0000000000000158 PMC4323546

[b32] Hwang T. L., Lee W. R., Hua S. C. & Fang J. Y. Cisplatin encapsulated in phosphatidylethanolamine liposomes enhances the *in vitro* cytotoxicity and *in vivo* intratumor drug accumulation against melanomas. Journal of dermatological science 46, 11–20 (2007). 17267180 10.1016/j.jdermsci.2006.12.011

[b33] Lippens S., Hoste E., Vandenabeele P., Agostinis P. & Declercq W. Cell death in the skin. Apoptosis 14, 549–569 (2009). 19221876 10.1007/s10495-009-0324-z

[b34] Luis A., Cruz C., Duarte A. P. & Domingues F. An alkenylresorcinol derivative from Hakea sericea fruits and their antimicrobial activity. Natural product communications 8, 1459–1462 (2013). 24354201

[b35] Armani C., Catalani E., Balbarini A., Bagnoli P. & Cervia D. Expression, pharmacology, and functional role of somatostatin receptor subtypes 1 and 2 in human macrophages. J Leukoc Biol 81, 845–855 (2007). 17148691 10.1189/jlb.0606417

[b36] Cervia D. *et al.* Cytotoxic effects and apoptotic signalling mechanisms of the sesquiterpenoid euplotin C, a secondary metabolite of the marine ciliate *Euplotes crassus*, in tumour cells. Apoptosis 11, 829–843 (2006). 16534550 10.1007/s10495-006-5700-3

[b37] Cervia D. *et al.* Molecular mechanisms of euplotin C-induced apoptosis: involvement of mitochondrial dysfunction, oxidative stress and proteases. Apoptosis 12, 1349–1363 (2007). 17440817 10.1007/s10495-007-0075-7

[b38] Perrotta C. *et al.* The thyroid hormone triiodothyronine controls macrophage maturation and functions: protective role during inflammation. Am J Pathol 184, 230–247 (2014). 24215914 10.1016/j.ajpath.2013.10.006

[b39] Cervia D. *et al.* The protein pheromone Er-1 of the ciliate Euplotes raikovi stimulates human T-cell activity: involvement of interleukin-2 system. Exp Cell Res 319, 56–67 (2013). 23103669 10.1016/j.yexcr.2012.10.007

[b40] Di Giuseppe G., Cervia D. & Vallesi A. Divergences in the Response to Ultraviolet Radiation Between Polar and Non-Polar Ciliated Protozoa: UV Radiation Effects in Euplotes. Microb Ecol 63, 334–338 (2011). 21904954 10.1007/s00248-011-9934-4

[b41] Perrotta C. *et al.* Syntaxin 4 is required for acid sphingomyelinase activity and apoptotic function. J Biol Chem 285, 40240–40251 (2010). 20956541 10.1074/jbc.M110.139287 PMC3001005

[b42] Perrotta C. *et al.* Nitric oxide boosts chemoimmunotherapy via inhibition of acid sphingomyelinase in a mouse model of melanoma. Cancer Res 67, 7559–7564 (2007). 17699758 10.1158/0008-5472.CAN-07-0309

[b43] Paolucci C., Rovere P., De Nadai C., Manfredi A. A. & Clementi E. Nitric oxide inhibits the tumor necrosis factor alpha -regulated endocytosis of human dendritic cells in a cyclic GMP-dependent way. J Biol Chem 275, 19638–19644 (2000). 10777484 10.1074/jbc.M000511200

[b44] Cazzato D. *et al.* Nitric oxide drives embryonic myogenesis in chicken through the upregulation of myogenic differentiation factors. Exp Cell Res 320, 269–280 (2014). 24240125 10.1016/j.yexcr.2013.11.006

[b45] Lievremont J. P. *et al.* The p75(NTR)-induced apoptotic program develops through a ceramide-caspase pathway negatively regulated by nitric oxide. J Biol Chem 274, 15466–15472 (1999). 10336437 10.1074/jbc.274.22.15466

[b46] De Palma C. *et al.* Deficient nitric oxide signalling impairs skeletal muscle growth and performance: involvement of mitochondrial dysregulation. Skelet Muscle 4, 22 (2014). 25530838 10.1186/s13395-014-0022-6 PMC4272808

[b47] Buono R. *et al.* Nitric oxide sustains long-term skeletal muscle regeneration by regulating fate of satellite cells via signaling pathways requiring Vangl2 and cyclic GMP. Stem Cells 30, 197–209 (2012). 22084027 10.1002/stem.783 PMC3378700

