# Supplementary material for: Climacostol reduces tumour progression in a mouse model of melanoma via the p53-dependent intrinsic apoptotic programme
Source: Sci Rep. 2016 Jun 7;6:27281. doi: 10.1038/srep27281 (PMC4895139; doi:10.1038/srep27281)
Supplement: Supplementary Information [file srep27281-s1.pdf]

Climacostol reduces tumour progression in a mouse model of melanoma via the p53-dependent intrinsic apoptotic programme

Cristiana Perrotta[1], Federico Buonanno[2], Silvia Zecchini[3], Alessio Giavazzi[3], Francesca Proietti Serafini[4], Elisabetta Catalani[4], Laura Guerra[4], Maria Cristina Belardinelli[4], Simona Picchietti[4], Anna Maria Fausto[4], Simone Giorgi[5], Enrico Marcantoni[5], Emilio Clementi[6,7], Claudio Ortenzi[2] & Davide Cervia[1,4]

[1]Department of Biomedical and Clinical Sciences "Luigi Sacco" (DIBIC), Università degli Studi di Milano, Italy

[2]Laboratory of Protistology and Biology Education, Department of Education, Cultural Heritage and Tourism, Università degli Studi di Macerata, Italy

[3]Unit of Clinical Pharmacology, University Hospital "Luigi Sacco"-ASST Fatebenefratelli Sacco, Milano, Italy

[4]Department for Innovation in Biological, Agro-food and Forest systems (DIBAF), Università degli Studi della Tuscia, Viterbo, Italy

[5]School of Sciences and Technologies, Section of Chemistry, Università degli Studi di Camerino, Italy

[6]Unit of Clinical Pharmacology, University Hospital "Luigi Sacco"-ASST Fatebenefratelli Sacco; National Research Council-Institute of Neuroscience; Department of Biomedical and Clinical Sciences "Luigi Sacco" (DIBIC), Università degli Studi di Milano, Milano, Italy

[7]Scientific Institute IRCCS Eugenio Medea, Bosisio Parini, Italy

*C. P. and F. B. contributed equally to this work.

Correspondence to: Prof. Davide Cervia Ph.D., Department for Innovation in Biological, Agro-food and Forest systems (DIBAF), largo dell'Università snc, Blocco D, Università degli Studi della Tuscia, 01100 Viterbo, Italy. Tel.: 39-0761-357040, e-mail: d.cervia@unitus.it

Supplementary Table S1. Primer pairs designed for real-time PCR analysis

Name	Gene symbol	Primer sequence
**p53**	*trp53*	F: 5’-CACGTACTCTCCTCCCCTCAAT-3’<br>R: 5’-AACTGCACAGGGCACGTCTT-3’
**p21**	*cdkn1a*	F: 5’-GGCCCGGAACATCTCAGG-3’<br>R: 5’-AAATCTGTCAGGCTGGTCTGC-3’
**Noxa**	*noxa1*	F: 5’-ACGCGAAGACTGGGACTCT-3’<br>R: 5’-AGCCCCTGTTAAAGTACATCCTA-3’
**Puma**	*bbc3*	F: 5’-GCTGAAGGACTCATGGTGAC-3’<br>R: 5’-CAAAGTGAAGGCGCACTG-3’
**Rpl32**	*rpl32*	F: 5’-TTAAGCGAAACTGGCGGAAAC-3’<br>R: 5’-TTGTTGCTCCCATAACCGATG-3’

F: forward, R: reverse

Supplementary Table S2. RNA duplex sequences of siRNA pools

p53	guide (5'-3') AGAUUUCAUUGUAGGUGCCCCC<br>guide (5'-3') AUACAAAUUUCCUUCCACCCCC<br>guide (5'-3') UACUUGUAGUGGAUGGUGGCCCCC<br><br>passenger (5'-3') GGGGGCACCUACAAUGAAAUCU<br>passenger (5'-3') GGGGGUGGAAGGAAAUUUGUAU<br>passenger (5'-3') GGGGGCCACCAUCCACUACAAGUA
Negative control	guide (5'-3') UUGUACUACACAAAAGUACCCCC<br>guide (5'-3') GAACGAAUUUAUAAGUGGCCCCC<br>guide (5'-3') ACAACAUUCAUAUAGCUGCCCCC<br><br>passenger (5'-3') GGGGGUACUUUUGUGUAGUACAA<br>passenger (5'-3') GGGGGCCACUUAUAAAUUCGUUC<br>passenger (5'-3') GGGGGCAGCUAUAUGAAUGUUGU
